# A Sex-Stratified Analysis of Monocyte Phenotypes Associated with HIV Infection in Uganda

**DOI:** 10.3390/v13112135

**Published:** 2021-10-22

**Authors:** Moises A. Huaman, Manuel G. Feria, Cissy Kityo, Sophie Nalukwago, Rashidah Nazzinda, David A. Zidar, Markella V. Zanni, Mark J. Siedner, Steven K. Grinspoon, Chris T. Longenecker

**Affiliations:** 1 Division of Infectious Diseases, Department of Medicine, University of Cincinnati College of Medicine, Cincinnati, OH 45229, USA; feriagmg@ucmail.uc.edu; 2 Joint Clinical Research Centre, Kampala P.O. Box 10005, Uganda; ckityo@jcrc.org.ug (C.K.); snalukwago@jcrc.org.ug (S.N.); rnazzinda@jcrc.org.ug (R.N.); 3 Division of Cardiology, Department of Medicine, Case Western Reserve University School of Medicine, Cleveland, OH 44106, USA; daz21@case.edu (D.A.Z.); cxl473@case.edu (C.T.L.); 4 Louis Stokes Cleveland Veterans Affairs Medical Center, Cleveland, OH 44106, USA; 5 Divisions of Infectious Diseases and Endocrinology, Department of Medicine, Massachusetts General Hospital, Boston, MA 02114, USA; mzanni@mgh.harvard.edu (M.V.Z.); MSIEDNER@mgh.harvard.edu (M.J.S.); sgrinspoon@mgh.harvard.edu (S.K.G.); 6 Harvard Medical School, Boston, MA 02115, USA; 7 University Hospitals Cleveland Medical Center, Cleveland, OH 44106, USA

**Keywords:** monocytes, inflammation, activation, HIV, women, sex

## Abstract

Women with HIV may experience higher rates of non-AIDS comorbidities compared to men with HIV, but the underlying mechanisms are not well understood. We investigated sex-related differences in the effects of HIV on monocyte phenotypes within the Ugandan Study of HIV effects on the Myocardium and Atherosclerosis (mUTIMA). Of 133 participants who provided blood for flow cytometry assays, 86 (65%) were women and 91 (68%) were persons living with HIV (PLWH) on antiretroviral therapy. The median age was 57 (interquartile range, 52–63) years. PLWH exhibited a lower proportion of circulating CD14^+^CD16^-^ classical monocytes (66.3% vs. 75.1%; *p* < 0.001), and higher proportion of CD14^+^CD16^+^ inflammatory monocytes (17% vs. 11.7%; *p* = 0.005) compared to HIV-uninfected participants. PLWH had an increased expression of the chemokine receptor CX3CR1 in total monocytes (CX3CR1^+^ monocytes, 24.5% vs. 4.7%; *p* < 0.001) and monocyte subsets. These findings were generally similar when analyzed by sex, with no significant interactions between sex and HIV status in adjusted models. Our data show that the inflammatory monocyte subset is expanded and monocyte CX3CR1 chemokine receptor expression is enhanced among PLWH, regardless of sex. Whether these parameters differentially affect risk for non-AIDS comorbidities and clinical outcomes in women with HIV requires additional investigation.

## 1. Introduction

Persons living with HIV (PLWH) have enhanced systemic inflammation and immune activation compared to the general population [1,2,3]. This persistent immune activation has been associated with increased morbidity and mortality due to noncommunicable diseases, including cardiovascular diseases, neurocognitive disorders, and non-AIDS cancers, even among PLWH with virologic suppression on antiretroviral therapy (ART) [2,4,5,6,7,8]. Studies have shown that women with HIV may experience disproportionately higher rates of non-AIDS comorbidities compared to men [9]; however, the underlying mechanisms driving these sex-related differences are not well defined. Due to the implication of monocyte alterations in the pathogenesis of non-AIDS comorbidities in PLWH [6,10], we analyzed potential sex differences in the phenotype of these immune cells.

Altered monocyte profiles and increased levels of monocyte activation markers have been reported in PLWH on long-standing HIV treatment [10]. Prior reports demonstrated that the proportion of CD16^+^ monocyte subsets is increased in PLWH, including CD14^+^CD16^+^ inflammatory and/or CD14^dim^CD16^+^ patrolling monocyte subsets [11,12,13]. Monocyte cell surface expression of activation markers and chemokine receptors is also altered in PLWH, but some of these parameters tend to normalize with ART [11]. Notably, most studies assessing relationships between HIV infection and monocyte activation have included participants in the United States or other developed countries, and have predominantly included men; therefore, results may not be representative of other settings such as sub-Saharan African (SSA) where HIV is more common among women and the epidemiology of potential confounders could differ [14]. Thus, our understanding of how sex-related differences may influence the effects of HIV infection on monocytes among individuals in SSA remains limited. In this study, we aimed to investigate potential sex differences in the effects of HIV on the phenotype of circulating monocytes within a cohort of participants with and without HIV, enrolled in the Ugandan Study of HIV effects on the Myocardium and Atherosclerosis (mUTIMA).

## 2. Materials and Methods

We analyzed cross-sectional data from a cohort of individuals enrolled in mUTIMA. Briefly, 100 PLWH and 100 persons without HIV (PWOH) are being observed in the mUTIMA project at the Joint Clinical Research Centre (JCRC) located in Kampala, Uganda. Inclusion criteria for all cohort participants included having a least 45 years of age and one cardiovascular disease risk factor such as hypertension, diabetes mellitus, dyslipidemia, tobacco use, or family history of heart disease. PLWH were recruited from the JCRC Clinic. PWOH were recruited from internal medicine clinics in Kampala, Uganda. History of hypertension and other medical conditions were collected by self-report and verified by review of available medical records. All PLWH who entered the cohort were required to have at least one HIV-1 viral load of <1000 copies/mL on stable ART within 6 months of study entry. Exclusion criteria were advanced kidney disease (estimated glomerular filtration rate [eGFR] <30 mL/min/1.73^2^), active immunosuppression, pregnancy, or uncontrolled inflammatory conditions. Participants presented to the JCRC for their year 2 follow-up visit between May 2017 and October 2019, which included blood sampling for flow cytometry. The study allowed for replacing cohort individuals who were lost to follow-up by the time of the year 2 visit with new participants of similar age and sex who met the same inclusion criteria. Samples utilized in this study were collected from participants when they presented for their year 2 visit. The total sample size of participants in the year 2 exam was 200; however, due to malfunctioning of the flow cytometer towards the end of the study, only a subset of this total sample had flow data available for this analysis. Using these data, we have previously published an analysis of the relationship between monocyte activation and latent tuberculosis infection [15]. The purpose of this secondary analysis was to explore sex differences in the HIV effects.

### 2.1. Monocyte Phenotyping

Fresh whole blood was collected in EDTA tubes for monocyte phenotyping using a MACSQuant^®^ flow cytometer at the JCRC as previously described [15]. Blood samples were processed for flow cytometry within ~4 h of collection at the JCRC Immunology Lab. Briefly, 300 μL of whole blood was added to 2 mL of Becton Dickinson (BD) red blood cell lysing solution on each tube, and incubated for 10 min following BD lysis buffer instructions. After washing the cells, antibodies were added into each tube and incubated for 30 min at 4 °C in FACS buffer. The antibodies in the monocyte panel included anti-CD14-pacific blue (M5E2, BD), anti-CD16-PE (3G8, BD), anti-CD62p-PE-Cy5 (AK-4, BD), anti-CD69-PE-Cy7 (L78, BD), anti-CX3CR1-APC (2A9-1, Biolegend), anti-HLA-DR-APC-Cy7 (L243, BD), and anti-tissue factor-FITC (VIC7, Sekisui). These markers were chosen to identify activated pro-inflammatory monocytes that have been associated with atherosclerosis or increased cardiovascular risk in people with and without HIV infection. CD14 and CD16 were used to define the three main subsets of interest as described below. P-selectin (CD62p) is involved in endothelial interactions that promote atherosclerosis and thrombosis [12]. CD69 assists lymphocyte trafficking into tissues and is associated with lipid alterations in PLWH [16], and it is considered an early activation marker. CX3CR1 is a chemokine receptor important in adhesion and migration to tissues, and it is also recognized as a monocyte activation marker associated with CVD, including carotid disease in PLWH [17]. HLA-DR is an activation marker that we have previously shown to be associated with latent tuberculosis infection [15]. Tissue factor expression on monocytes is elevated in PLWH and people with acute coronary syndrome [12] and may be reduced by statin therapy [18].

**Figure 1:**
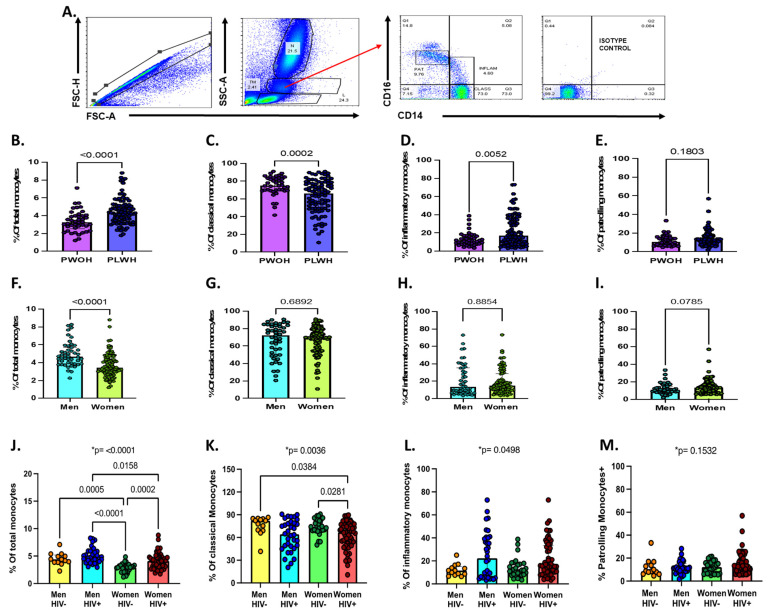
Total monocytes and monocyte subsets by sex, HIV status, and sex/HIV status. (**A**) Gating strategy for total and monocyte subsets. (**B**) Percentage of total monocytes from whole blood by HIV status. (**C**–**E**) Percentage of classical, inflammatory, and patrolling monocyte subsets by HIV status. (**F**) Percentage of total monocytes by sex. (**G**–**I**) Percentage of monocyte subsets by sex. Statistical differences across two groups were assessed using Mann–Whitney test for panels B through I. (**J**) Percentage of total monocytes from whole blood by sex and HIV status. (**K**–**M**) Percentage of monocyte classical, inflammatory, and patrolling monocyte subsets by sex and HIV status. Statistical differences across the four groups were assessing using Kruskal–Wallis test for panels J through M (* *p*-value show on the top). In addition, Dunn’s multiple comparisons test was used to test for differences between specific groups (indicated in brackets). A *p*-value < 0.05 was considered statistically significant. Non-significant *p*-values on Dunn’s multiple tests are not shown.

Flow cytometry standard (FCS) files were stored for further analyses in FlowJo v10 software. Total monocytes were identified based on cell size and granularity in FSC-H vs. FSC-A plots (see Figure 1A). Three monocyte subsets were defined based on cell surface CD14 and CD16 expression as previously described [12,19]: CD14^+^CD16^-^ classical monocytes, CD14^+^CD16^+^ inflammatory (intermediate) monocytes, and CD14^dim^CD16^+^ patrolling (non-classical) monocytes. Isotype controls were used to define positive gates (Supplementary Figure S1). Our gating strategy was validated by two independent team members using a subset of FCS study files, demonstrating good reproducibility with a median (Q1-Q3) intraclass correlation coefficient of 0.965 (0.940–0.993) for the 20 monocyte subsets. For the primary analyses, cell surface marker expression was defined as a proportion. Given the significant findings for CX3CR1, we also performed an analysis using median fluorescence intensity, a quantitative measure of the amount of surface marker expression on cells.

### 2.2. Statistical Analyses

Study data were entered in REDCap [20]. Flow cytometry data analyzed in FlowJo were exported into MS Excel and then transferred to REDCap. We used Kruskal–Wallis and Dunn tests for multiple group comparisons of flow cytometry data. We used simple and multiple linear regression models for unadjusted and adjusted analyses, respectively. These models included log(10)-transformed flow cytometry data as the dependent variable, and HIV status as the main independent variable. Covariates included in adjusted analyses were age in years, sex at birth (henceforth referred to as sex), history of diabetes mellitus (DM), total cholesterol in mg/dL, body mass index (BMI), waist-to-hip ratio, and history of prior or latent TB. Models were checked for collinearity using variance inflation factors (VIFs). Due to our interest in potential sex-related differences in the effects of HIV on monocyte parameters, we tested all models for HIV by sex interactions. Regression results are presented as b-coefficients, accompanied by their 95% confidence interval (95% CI). The b-coefficients for HIV status represent the degree of change in the log-transformed percentage value of the cell surface marker expression in PLWH compared to PWOH. The b-coefficients for sex represent the degree of change in the log-transformed percentage value of the cell surface marker expression in females compared to males. For adjusted b-coefficients, the results were adjusted by all co-variates included in the models. A heat map was designed with the percentage of cells expressing the surface markers of interest on total monocytes and monocyte subsets, stratified by sex and HIV status. We used Stata v12 (College Station, TX), R v 4.1.0 software (R Foundation), and Prism v 9.2.0 (GraphPad Software, San Diego, CA) for data analyses and generation of graphics.

## 3. Results

Data from 133 participants with available flow cytometry data were included in this analysis. This included data from 86 (65%) women and 47 (35%) men. Among the women, 57 (66%) were PLWH. Among the males, 34 (72%) were PLWH. Table 1 shows the characteristics of the study population by HIV and sex at birth. Age, diabetes mellitus, and hypertension were similar across the groups. Tobacco use was uncommon within our cohort, with only 3 participants who reported active tobacco use at time of their visit—all of them were men with HIV. Women with HIV had the highest total cholesterol levels (*p* = 0.017), with low-density lipoprotein (LDL) and high-density lipoprotein (HDL) cholesterol fractions following similar trends. In terms of anthropometric characteristics, we observed differences in BMI by sex and HIV status (*p* < 0.001), with women without HIV having the highest BMIs and men with HIV having the lowest BMIs. Waist-to-hip ratio was overall lower in women compared to men (*p* < 0.001), without significant differences by HIV status. There were no participants who reported having viral hepatitis B or hepatitis C infection. Of the 133 participants, 125 had a defined tuberculosis (TB) status as previously described [15]. Overall, latent TB or prior history of active TB was common (54%), with the highest rates among women without HIV.

Our gating strategy and description of monocyte subsets by sex or HIV status is shown in Figure 1. Overall, we observed a higher proportion of total monocytes (4.3 [interquartile range (IQR), 3.4–5.2] vs. 3.2 [2.6 vs. 3.9]; *p* < 0.001; Figure 1B), lower proportions of classical monocytes (66.3 [IQR, 49.3–77.8] vs. 75.1 [IQR, 69.3–83.5]; *p* < 0.001; Figure 1C) and higher proportions of inflammatory monocytes (17 [IQR, 8.8–36.3] vs. 11.7 [IQR, 8.1 vs. 16.4]; *p* = 0.006; Figure 1D) in PLWH compared to PWOH. No differences in the proportion of patrolling monocytes by HIV status were noted (Figure 1E). Women had lower proportions of circulating monocytes compared to men (3.4 [IQR, 2.8–4.5] vs. 4.7 [IQR, 3.9–5.7] vs.; *p* < 0.001; Figure 1F), but no significant differences were found in the proportion of monocyte subsets by sex (Figure 1G–I).

Among women, those with HIV had higher proportions of total monocytes compared to those without HIV (4.1 [IQR, 3.1–4.8] vs. 2.9 [IQR, 2.2–3.3]; *p* < 0.001; Figure 1J). There were differences in the proportions of CD14^+^CD16^-^ classical monocytes (*p* = 0.0036) and CD14^+^CD16^+^ inflammatory monocytes (*p* = 0.049) by sex and HIV status (Figure 1K,L). No significant differences were found in the proportion of CD14^dim^CD16^+^ patrolling monocytes by sex and HIV status (Figure 1M).

**Figure 2:**
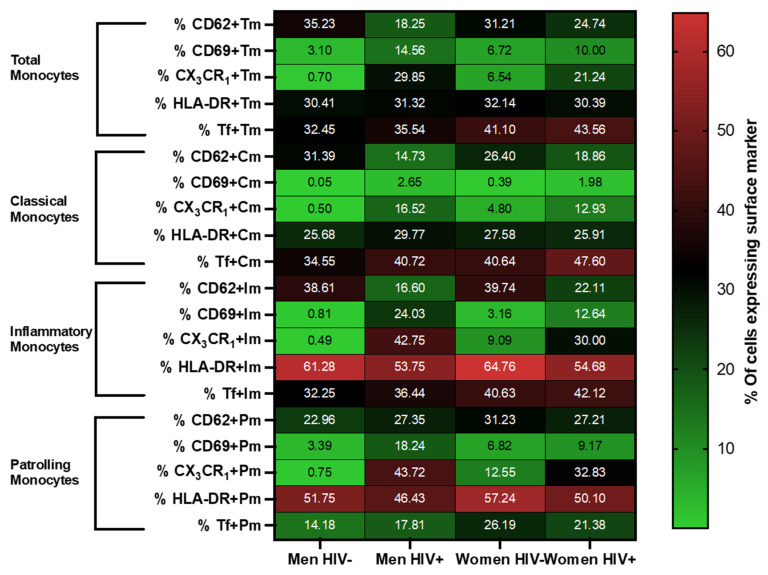
Monocyte phenotyping by sex/HIV status. Heat map depicting the percentage of expression of CD62p, CD69, CX3CR1, HLA-DR, and tissue factor in total, classical, inflammatory, and patrolling monocytes (Y axis) by sex/HIV group (X axis). The numbers indicated within each box are the average percentage of cells expressing the surface marker indicated in the Y axis, for the given sex/HIV group indicated in the X axis.

Surface expression of CD62p, CD69, CX3CR1, HLA-DR, and tissue factor were investigated on total monocytes and monocyte subsets by sex and HIV status (Figure 2). Table 2 shows the results of regression models of CX3CR1 and CD62p expression for HIV status. Regression models for CD69 and HLA-DR are not shown since there were no significant differences by HIV status.

In terms of HIV status effects, we found higher proportions of CX3CR1 expression on monocytes of PLWH compared to PWOH, even after accounting for potential confounders in adjusted regression models (*p* = 0.013). Comparable trends of higher CX3CR1 expression in PLWH were observed within the classical, inflammatory, and patrolling monocyte subsets (Table 2). In models that used CX3CR1 median fluorescence intensity values (log-transformed MFI) as the dependent variable, we found that CX3CR1 density was also higher among PLWH compared to PWOH (unadjusted b = +0.41; [95% CI, 0.17–0.65]; *p* < 0.001; adjusted b = +0.37; [95% CI, 0.10–0.64]; *p* = 0.008). For CD62p, we observed a decreased proportion of CD62p^+^ patrolling monocytes in PLWH compared to PWOH in adjusted models (*p* = 0.028).

In contrast to HIV status, there were no statistically significant sex effects on surface expression of CD62p or CX3CR1 in unadjusted or adjusted models among pooled PLWH and PWOH participants (Supplementary Table S1). Furthermore, the HIV effects described above were generally similar when analyzed by sex, with no significant interactions between sex and HIV status in any of the adjusted models (all *p* for interaction > 0.05, Supplementary Table S1).

## 4. Discussion

In this study, conducted among middle- and older-age women and men living in sub-Saharan Africa, we found that PLWH have an expanded proportion of inflammatory monocytes compared to PWOH. Additionally, among PLWH vs. PWOH, across total monocytes and all monocyte subsets, there was an increased percentage of cells expressing CX3CR1 and an increased density of expression of CX3CR1. These findings were generally similar when analyzed by sex and remained consistent after adjusting for multiple potential confounders including age, BMI, waist-to-hip ratio, and history of either DM or prior/latent TB.

We found that women had lower proportions of total monocytes than men within PLWH and PWOH groups. These results are in line with prior studies in the general population, which showed that women tend to have lower monocyte counts and percentages compared to men across age groups [21,22]. The pathophysiologic consequences of these differences are not well defined, but have led to studies like ours focused on profiling monocytes and their subsets with particular attention on potential sex dissimilarities.

As previously reported by our group and others [12,15], PLWH had a higher proportion of inflammatory monocytes and lower classical monocytes than controls without HIV. Although total monocyte proportions were overall lower among women compared to men, we found no significant sex-related differences in the association between HIV infection and the inflammatory monocyte subset. Inflammatory monocytes are recognized for their robust pro-inflammatory responses, including production of cytokines such as tumor necrosis factor-alpha and interleukin-6 upon stimulation [23,24]. An expansion of inflammatory monocytes has been described in other inflammatory conditions beyond HIV, including autoimmune disorders [25] and cardiovascular disease [12]. Our data indicate that women with HIV share this phenotype, similar to what has been observed in other male-predominant cohorts of PLWH [12]. Our results are consistent with other reports of significant alterations in monocyte subsets among women with HIV from developed countries [26].

We found that PLWH had an increased expression and density of CX3CR1 on total monocytes as well as across the three monocyte subsets. The CX3CR1 chemokine receptor is involved in adhesion and migration of monocytes into the tissues, and has an important role in atherogenesis and plaque development [27,28]. Whether CX3CR1 expression is enhanced in PWLH on chronic ART has been under debate. A prior study showed increased expression of CX3CR1 monocytes of PLWH on ART compared to HIV-uninfected controls [29]. However, other studies reported either decreased CX3CR1 expression [13], normalization of levels with ART initiation [11], or no differences with HIV-uninfected controls after adjusting for certain demographic and lifestyle parameters [30]. We observed increased CX3CR1 expression across monocyte subsets in ART-treated PLWH living in Uganda, after accounting for several potential confounders. The implications of this finding may be clinically relevant, as prior studies have associated augmented expression of CX3CR1 on monocytes to increased carotid atherosclerosis [17], neuroinflammation [30], and aging [31].

Interestingly, we found a decreased expression of of CD62p (P-selectin) on the surface of inflammatory monocytes, but not on total monocytes or the other monocyte subsets. A prior study showed that PLWH had increased expression of CD62p on monocytes [12]. Our finding may indicate a compensatory feedback mechanism in pro-inflammatory, pro-thrombotic states [32]. Future studies are needed to further investigate this finding and its potential clinical consequences.

Noncommunicable diseases have become leading causes of morbidity and mortality among women and men worldwide [33]. Sex-specific differences in biological and behavioral risk factors as well as treatment disparities have been described, with wide variations across regions [34]. Furthermore, studies suggest that women are at increased risk of complications and poorer outcomes from noncommunicable diseases such as cardiovascular disease and diabetes mellitus, compared to their male counterparts [35,36]. Studies also suggest that women with HIV experience disproportionately higher rates of non-AIDS comorbidities compared to men with HIV [9]. The underlying mechanisms driving these sex-related differences are not well understood. Residual monocyte activation is an important contributor in the pathogenesis of non-AIDS comorbidities in PLWH on chronic antiretroviral therapy ART [6,10,37,38]. In our study, we found an expansion of inflammatory monocytes and increased CX3CR1 expression across monocyte subsets in both women and men with HIV, suggesting similar alterations in monocyte phenotypes regardless of sex. Our study adds to the growing literature indicating increased monocyte activation among women with HIV. Compared to women without HIV, women with HIV have increased monocyte activation markers, including higher cell surface expression of the chemokine receptor CCR2, soluble CD14, soluble CD163, and CCL2 levels [39]. It is possible that similar levels of monocyte activation could differentially affect risk for non-AIDS comorbidities and clinical outcomes in women versus men with HIV, given known biological and socio-behavioral factors unique to women [40]. For instance, enhanced type 1 interferon production by innate cells may be mediated by estrogen receptor signaling in women [41]. The degree to which immune mechanisms contribute to the higher non-AIDS comorbidity burden in women with HIV, especially in the sub-Saharan African context, merits further investigation.

Our study had limitations. We could only include a selected number of markers in our monocyte panel as allowed by the flow cytometer utilized in this study. Future studies may include additional biologic markers of interest to have a more in-depth profiling of monocytes, and include in vitro stimulation assays to assess their pro-inflammatory responses. However, our in-country setup allowed building local capacity and processing fresh blood specimens on the same day of sample collection, thus avoiding potential cell disturbances introduced by freezing and thawing procedures. Although ours is one of the largest studies of monocyte profiling of PLWH in sub-Saharan Africa, we recognize that our analysis by sex and HIV status may not have been powered to detect small differences across some of the groups (for instance, the male PLWH group only included 13 individuals). Nevertheless, we were able to detect a clear association between HIV, inflammatory monocytes, and CX3CR1 expression on total monocytes and subsets even after adjusting for important confounders. Furthermore, about two thirds of our population were women, a group that has been traditionally underrepresented in HIV research, particularly in immunologic studies such as ours. Other than TB status, information on coinfections was limited to self-report, possibly underestimating the true co-pathogen burden in this population. All participants with HIV were on ART for a median of ~13 years and ~90% were virologically suppressed, therefore it is unlikely that undiagnosed active opportunistic infections were driving the observed differences in monocyte parameters between PLWH and PWOH. Furthermore, the inclusion of PLWH on long-standing ART makes our results relevant to the overall aging HIV population at risk for non-AIDS comorbidities.

## 5. Conclusions

In conclusion, our data show that the inflammatory monocyte subset and CX3CR1 receptor expression are expanded in PLWH in Uganda. These findings were generally similar when analyzed by sex, with no significant interactions between sex and HIV status in adjusted models. Whether these cells differentially affect risk for non-AIDS comorbidities and clinical outcomes in women with HIV requires additional investigation.

## Figures and Tables

**Table 1:** Characteristics of the study population by sex and HIV status.

	All(*n* = 133)	Men		Women		*p*-Value
		PWOH *n* = 13	PLWH *n* = 34	PWOH *n* = 29	PLWH *n* = 57	
**Traditional Cardiometabolic Risk**						
Age in years	57 (52–63)	57 (52–63)	62 (50–69)	55 (52–62)	56 (52–61)	0.325
Diabetes mellitus	42 (32)	7 (54)	10 (29)	11 (38)	14 (25)	0.179
Cardiovascular disease	5 (4)	1 (8)	2 (6)	1 (4)	1 (2)	0.366
Hypertension	115 (87)	9 (69)	29 (85)	26 (90)	51 (89)	0.258
Tobacco use at time of visit	3 (2)	0	3 (9)	0	0	0.177
Body mass index (kg/m^2^)	28.4 (24.5–32.5)	28.4 (26.8–30.6)	24.4 (21.8–26.8)	31.8 (28.4–33.9)	28.8 (25.6–34.9)	<0.001
Waist-to-hip ratio	0.91 (0.86–0.95)	0.96 (0.95–1)	0.94 (0.91–0.98)	0.87 (0.83–0.90)	0.88 (0.83–0.92)	<0.001
Total cholesterol (mg/dL)	204 (173–231)	182 (163–205)	192 (165–226)	194 (173–231)	217 (189–240)	0.017
LDL cholesterol (mg/dL)	129 (107–152)	119 (101–138)	113 (86–135)	133 (112–168)	136 (115–158)	0.021
HDL cholesterol (mg/dL)	52 (43–62)	45 (42–52)	51 (40–66)	49 (43–63)	56 (49–74)	0.034
**Coinfections**						
Latent TB or prior TB	68 (54)	6 (46)	18 (58)	19 (65)	25 (46)	<0.001
**HIV Characteristics**						
HIV Viral load ≤ 20 copies/mL	78 (87)	N/A	32 (94)	N/A	46 (82)	0.124
Nadir CD4 count	161 (65–297)	N/A	172 (77–287)	N/A	150 (64–297)	0.646
HIV duration (years)	13.9 (11.7–15.2)	N/A	14 (11.7–15.8)	N/A	13.9 (11.9–14.5)	0.261
ART duration (years)	12. 7 (10.1–14)	N/A	12.7 (10.7–14.7)	N/A	12.6 (10.1–14)	0.382
PI use at time of visit	23 (25)	N/A	7 (21)	N/A	16 (28)	0.427
INSTI use at time of visit	2 (2)	N/A	2 (6)	N/A	0	0.137

Continuous variables are presented as median (interquartile range) and categorical variables as n (%). Abbreviations: people without HIV (PWOH), people living with HIV (PLWH), interquartile range (IQR), low-density lipoprotein (LDL), high-density lipoprotein (HDL), antiretroviral therapy (ART), protease inhibitor (PI), and integrase inhibitor (INSTI).

**Table 2:** Association between HIV infection and total monocytes, monocyte subsets, and surface expression of CX3CR1 and CD62p.

	Unadjusted B-Coefficient ^a^	*p*-Value	Adjusted B-Coefficient ^b^	*p*-Value
**Monocyte Subsets**				
Total monocytes	0.13 (0.08–0.19)	<0.001	0.11 (0.07–0.17)	<0.001
Classical monocytes	−0.10 (−0.15–−0.05)	<0.001	−0.09 (−0.15–−0.03)	0.005
Inflammatory monocytes	0.17 (0.05–0.29)	0.006	0.18 (0.04–0.31)	0.012
Patrolling monocytes	0.06 (−0.03–0.14)	0.174	0.03 (−0.06–0.12)	0.516
**Proportion of CX3CR1+ Monocytes**				
CX3CR1 in total monocytes	0.68 (0.26–1.10)	0.002	0.59 (0.13–1.07)	0.013
CX3CR1 in classical monocytes	0.99 (0.45–1.55)	<0.001	0.91 (0.30–1.52)	0.004
CX3CR1 in inflammatory monocytes	0.94 (0.44–1.44)	<0.001	0.78 (0.22–1.33)	0.007
CX3CR1 in patrolling monocytes	0.55 (0.03–1.07)	0.038	0.49 (−0.11–1.08)	0.109
**Proportion of CD62p+ Monocytes**				
CD62p in total monocytes	−0.12 (−0.38–0.14)	0.357	−0.05 (−0.33–0.25)	0.761
CD62p in classical monocytes	−0.28 (−0.69–0.14)	0.187	−0.14 (−0.60–0.33)	0.560
CD62p in inflammatory monocytes	−0.81 (−1.23–−0.38)	<0.001	−0.53 (−1.00–−0.06)	0.028
CD62p in patrolling monocytes	0.09 (−0.20–0.39)	0.519	0.17 (−0.17–0.51)	0.319

^a^ B-coefficient of linear regression using log-transformed values of monocyte proportions as the dependent variable, and HIV status as the only independent variable. ^b^ B-coefficient of linear regression using log-transformed values of monocyte proportions as the dependent variable, and HIV status as the main independent variable adjusted for sex at birth, age in years, history of diabetes mellitus, total cholesterol in mg/dL, body mass index, waist-to-hip ratio, and latent or prior tuberculosis.

## Data Availability

Data used in this study are available from the authors upon reasonable request.

## Supplementary Materials

The following are available online at https://www.mdpi.com/article/10.3390/v13112135/s1, Figure S1: Gating strategy, Table S1: Association between sex and total monocytes, monocyte subsets, and surface expression of CX3CR1 and CD62p.
