# Supplementary material for: A Sex-Stratified Analysis of Monocyte Phenotypes Associated with HIV Infection in Uganda
Source: Viruses. 2021 Oct 22;13(11):2135. doi: 10.3390/v13112135 (PMC8620269; doi:10.3390/v13112135)
Supplement: Supplementary file 1 [file viruses-13-02135-s001.zip › viruses-1384304-supplementary.pdf]

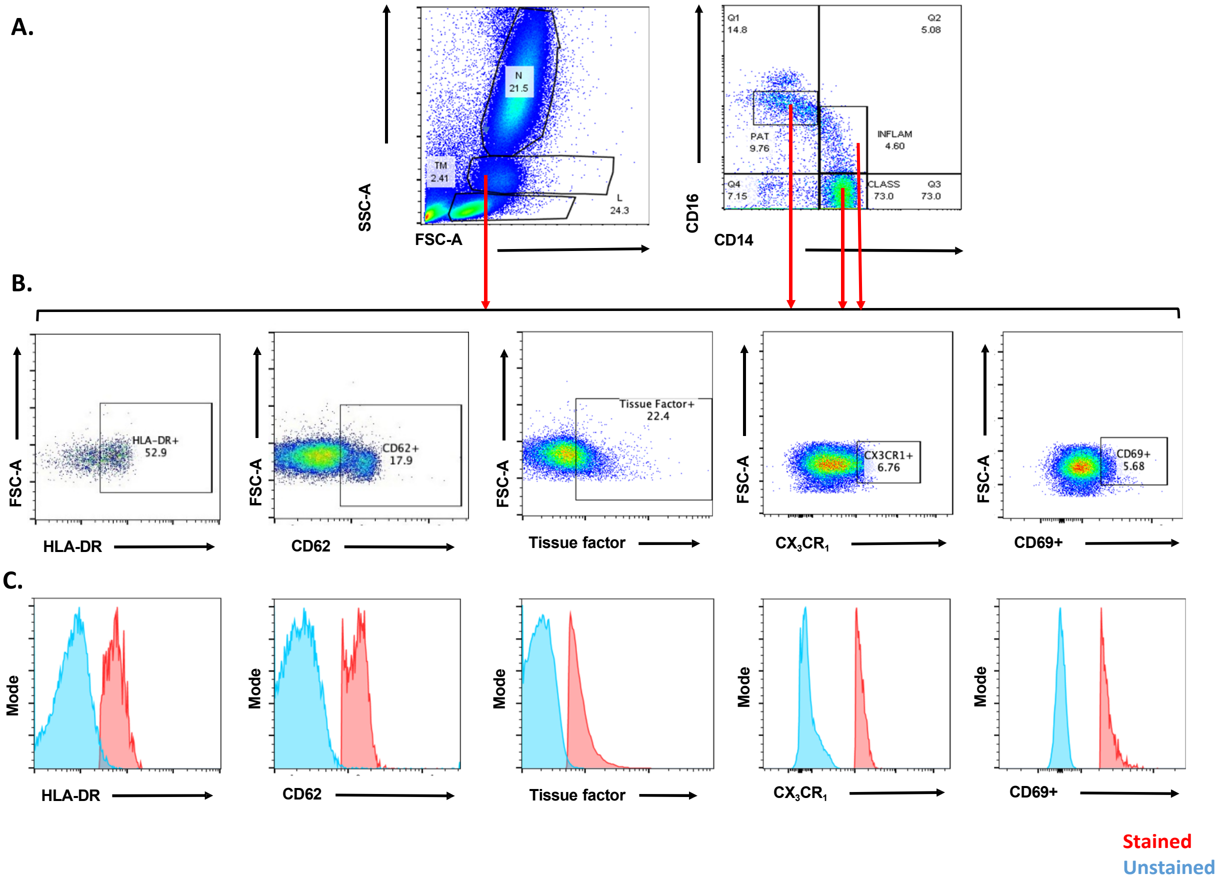

Figure S1. Gating strategy. **(A)** Gating strategy for total monocytes and monocyte subsets (classical, inflammatory, and patrolling monocytes). **(B)** Pseudo-color plots derived from the total monocytes and subsets monocytes for surface markers included in the study (HLA-DR, CD62, Tissue factor, CX3CR1, and CD69). **(C)** Histograms of each marker showing unstained cells (blue histogram) vs. specific antibody staining (Red histogram). FSC: forward scatter; SSC: side scatter.

Table S1. Association between sex and total monocytes, monocyte subsets, and surface expression of CX3CR1 and CD62p.

	Unadjusted *b* coefficient [a]	*P* value	Adjusted *b* coefficient [b]	*P* value	HIV*Sex *P* value [c]
Monocyte subsets					
Total monocytes	-0.14 (-0.19 – -0.08)	<0.001	-0.14 (-0.21 – -0.08)	<0.001	0.115
Classical monocytes	0.01 (-0.05 – 0.06)	0.854	0.01 (-0.05 – 0.08)	0.685	0.908
Inflammatory monocytes	-0.01 (-0.12 – 0.12)	0.994	-0.05 (-0.21 – 0.11)	0.524	0.833
Patrolling monocytes	0.07 (-0.01 – 0.15)	0.070	0.03 (-0.08 – 0.13)	0.598	0.604
Proportion of CX3CR1+ monocytes					
CX3CR1 in total monocytes	0.05 (-0.38 – 0.47)	0.827	0.06 (-0.47 – 0.59)	0.811	0.736
CX3CR1 in classical monocytes	-0.22 (-0.78 – 0.33)	0.429	-0.42 (-1.17 – 0.32)	0.265	0.242
CX3CR1 in inflammatory monocytes	-0.06 (-0.56 – 0.45)	0.822	0.06 (-0.62 – 0.74)	0.858	0.299
CX3CR1 in patrolling monocytes	-0.02 (-0.53 – 0.50)	0.952	0.31 (-0.39 – 1.0)	0.388	0.092
Proportion of CD62p+ monocytes					
CD62p in total monocytes	0.14 (-0.11 – 0.39)	0.284	0.30 (-0.03 – 0.62)	0.075	0.839
CD62p in classical monocytes	-0.05 (-0.48 – 0.37)	0.809	0.19 (-0.34 – 0.73)	0.480	0.548
CD62p in inflammatory monocytes	0.35 (-0.09 – 0.79)	0.118	0.48 (-0.06 – 1.0)	0.079	0.848
CD62p in patrolling monocytes	0.01 (-0.28 – 0.30)	0.955	0.16 (-0.22 – 0.55)	0.405	0.679

[a] *b* coefficient of linear regression using log-transformed values of monocyte proportions as the dependent variable, and sex as the only independent variable.

[b] *b* coefficient of linear regression using log-transformed values of monocyte proportions as the dependent variable, and sex as the main independent variable adjusted HIV status, age in years, history of diabetes mellitus, total cholesterol in mg/dL, body mass index, waist-to-hip ratio, and latent or prior tuberculosis.

[c] P value for the HIV*sex interaction term when added to the full adjusted model.
